# Strength Characteristics of Alkali-Activated Slag Mortars with the Addition of PET Flakes

**DOI:** 10.3390/ma14216274

**Published:** 2021-10-21

**Authors:** Agnieszka Kocot, Andrzej Ćwirzeń, Tomasz Ponikiewski, Jacek Katzer

**Affiliations:** 1 Department of Building Materials and Processes Engineering, Silesian University of Technology, 44-100 Gliwice, Poland; a.koziolek@onet.eu (A.K.); tomasz.ponikiewski@polsl.pl (T.P.); 2 Department of Civil, Environmental and Natural Resources Engineering, Luleå University of Technology, 971 87 Luleå, Sweden; andrzej.cwirzen@ltu.se; 3 Institute of Geodesy and Civil Engineering, Faculty of Geoengineering, University of Warmia and Mazury in Olsztyn, 10-719 Olsztyn, Poland

**Keywords:** ground granulated blast furnace slag, PET, mortar, geopolymer, waste management

## Abstract

The production of ordinary Portland cement is associated with significant CO_2_ emissions. To limit these emissions, new binders are needed that can be efficiently substituted for cement. Alkali-activated slag composites are one such possible binder solution. The research programme presented herein focused on the creation of alkali-activated slag composites with the addition of PET flakes as a partial substitute (5%) for natural aggregate. Such composites have a significantly lower impact in terms of CO_2_ emissions in comparison to ordinary concrete. The created composites were differentiated by the amount of activator (10 and 20 wt.%) and curing temperature (from 20 to 80 °C). Their mechanical properties were tested, and a scanning electron microscope analysis was conducted. Compressive and flexural strengths ranging from 29.3 to 68.4 MPa and from 3.5 to 6.1 MPa, respectively, were achieved. The mechanical test results confirmed that a higher amount of activator improved the mechanical properties. However, the influence of the PET particles on the mechanical properties and microstructure varied with the curing temperature and amount of activator. Areas that require further research were identified.

## 1. Introduction

In 2018, the global production of cement exceeded 4000 Mt. Cement production is the third greatest source of global CO_2_ emissions. Approximately 1.5 Gt of carbon dioxide is created each year during the manufacture of Portland cement. This constitutes about 4% of global CO_2_ emissions from fossil fuels. To put that in perspective, Sweden emitted about 1.5 Mt of CO_2_, China emitted 782 Mt of CO_2_, and India emitted more than 125 Mt CO_2_ during the process of cement production. In 2018, Poland alone emitted 7.0 Mt of CO_2_ associated with Portland cement production [1]. Global Portland cement production was estimated at 4050 Mt in that year. China produced 2200 Mt, constituting over half of global cement production, while India produced 300 Mt [2]. Swedish and Polish cement production reached around 3 Mt and 19 Mt, respectively [3,4]. At the same time, 3.7 Mt of blast furnace slag and 8.2 Mt of fly ash compounds were generated in Poland alone [5]. The production of ordinary Portland cement (OPC) requires a significant amount of energy to heat the raw materials and then grind them to obtain powdered clinker. Therefore, substitution of OPC with alternative binders [6] could significantly lower the carbon footprint of concrete [6]. Such binders have been developed over the last few decades, and binders activated with alkali solutions seem to be the most promising [7]. Alkali-activated material (AAM) refers to alumino-silicate-based binders; the most common are blast furnace slag (BFS), fly ash (FA), and natural pozzolans. Geopolymers are a subgroup of AAM [7]. Ground granulated blast furnace slag (GGBFS), which is typically used as a pozzolanic material added to Portland-cement-based systems, can also be activated with strong alkalis. The properties of slag concrete depend on the chemical composition of the slag, the activator used, curing conditions, etc. [8,9]. GGBFS-based concrete is more resistant to fire, acid solutions, and corrosion in comparison to ordinary concrete [10,11]. However, it is also characterised by a higher shrinkage than OPC. Tests conducted by Patel and Shah [12] proved that concrete based on GGBFS activated by sodium silicate and sodium hydroxide (molarity of NaOH equal to 12 M) and cured at temperatures above 60 °C achieves higher compressive strength (by approximately 18%) in comparison to concrete cured at ambient temperature. The absorption of water in the case of GGBFS mortar was lower in comparison to a metakaolin-based sample. This result was related to the denser microstructure of mortar with GGBFS. GGBFS performed better than OPC in terms of mechanical properties when exposed to high temperatures of up to 800 °C after 28 days of curing [13].

The most common activator for GGBFS is silicate in the form of solution or powder [6]. The product of GGBFS activated with silicate solution is poorly crystalline calcium-alumino-silicate-hydrate (CASH) gel. Binders containing a high concentration of MgO have higher strength than GGBFS with its decreased content of magnesium oxide. Increasing the Si/Al ratio influences the compressive strength of geopolymer concrete [14]. When GGBFS is activated with sodium silicate, CSH is not the major product of hydration. Hydrotalcite (Mg_6_Al_2_CO_3_(OH)_16_·4H_2_O) appears to be one of the products of hydration when the binder is activated with NaOH. Hydrotalcite is described as small crystals appearing near the CSH gel [15], and it appears in GGBFS binders with high MgO content (MgO > 5%) [6]. One should also remember that the pore network is refined by increasing the dosage of the activator, [6]. The morphology and chemical composition of the composite do not change significantly when the amount of activator changes [16]. Using GGBFS as a binder is beneficial for the environment, mostly because it reduces the demand for cement. It is also an effective way to dispose of by-products. However, it should be noted that GGBFS requires an activator that is based on chemicals, which also need to be produced and can be hazardous for the environment and health.

The disposal of plastic waste is a great challenge for present and future generations. For example, ineffective procedures have caused a phenomenon called “microplastic soup” in the Indian Ocean. Flaked artificial wastes floating in the ocean constitute a serious threat to the environment. The concentration of particles of different size was found to be 34,000 pieces per square kilometre [17]. For many countries such as Sri Lanka, Jordan, or India, plastic waste is a catastrophic environmental problem [18,19].

Recently, plastic waste has started to be used as a replacement for natural aggregates in concrete production to reduce the amount of waste disposed to landfills. In the Philippines, plastic bags were shredded and reused to produce “eco-bricks”. This building material was used for to construct pavements, a clock tower, and a canteen in a school [20]. Using plastic waste as a replacement for aggregates in ordinary concrete appears to be quite challenging. For example, replacing natural aggregates with PET particles decreased the compressive strength and lowered the durability [21]. Selected examples of studies on the application of PET particles as a replacement for natural aggregate are presented in Table 1.

PET particles, apart from traditional cementitious composites, have also been used in the production of alkali-activated slag mortars [28]. It was demonstrated that replacing high volumes of natural aggregate (above 20%) significantly influences the compressive strength of the hardened composite. Thus, it was decided to keep the amount of added PET particles reasonably small (5%) to maintain the mechanical characteristics of the alkali-activated composites. The research programme presented herein, tested the mechanical properties of alkali-activated composites based on GGBFS and cured at different temperatures. Some of the composites were modified by the addition of PET flakes. The achieved results are compared and discussed.

## 2. Materials and Methods

**Figure 1:**
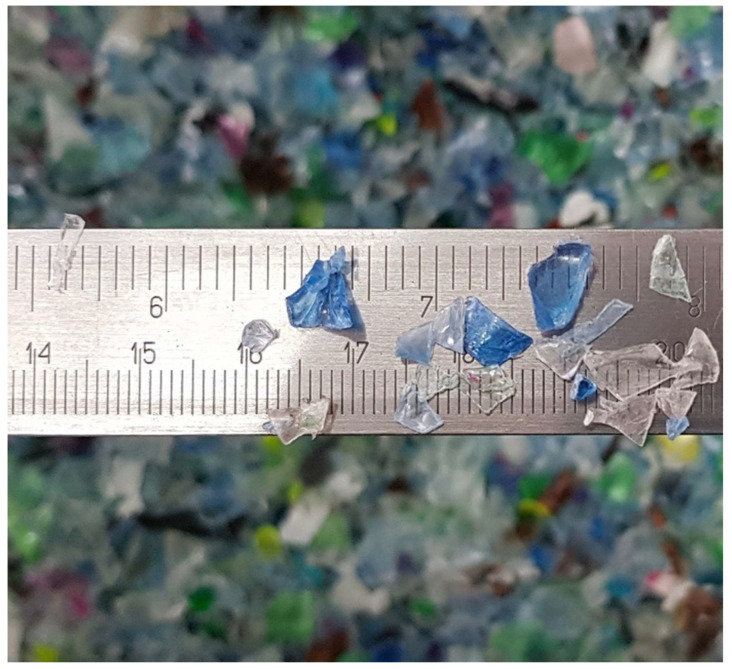
PET flakes.

The PET flakes used in the study were obtained from a local recycling company (Argo, Poland). They were produced from plastic bottles through shredding. The PET flakes delivered to the laboratory were washed. The flake size ranged from 0 mm to 16 mm (see Figure 1). Flakes were sieved and divided into two fractions containing particles of 0 mm to 4 mm and those of 4 mm to 16 mm; only the first group was used in this research.

Quartz sand with a maximum particle diameter of 2 mm was used. The sand was characterised by a specific gravity of 2.65 g/cm^3^. GGBFS with a dry bulk density of 150 kg/m^3^, sourced from Merox (Sweden), was utilised as a binder. Its chemical composition is shown in Table 2.

A mix of sodium hydroxide (NaOH) and sodium silicate (Na_2_SiO_3_) was used as the alkali activator. The crystalline NaOH (purity of 98%) was characterised by a SiO_2_/Na_2_O ratio of 2.2 and solid content of 49.97 wt. %. The silicate-to-sodium (SiO_2_/Na_2_O) ratio of the prepared solution was 1.5 and was set after considering earlier results [16].

Five mixes contained 10 wt.% of the alkali activator, and four mixes used 20 wt.% of the activator. The amount of activator was calculated as a weight percentage of the total binder amount. PET flakes were used to replace 5 wt. % of the fine aggregates. The aggregate-to-binder ratio was 3:1 for all mixes. A water/binder ratio of 0.46 was chosen based on initial test results. The workability of the fresh mortars was assessed using the slump flow method (UNE EN 1015-3:2000/A2:2007) and was found to be 141 mm. The amount of water originating from the alkali activator was considered when calculating the water-to-cement ratio. In total, eight testing mixes and one reference mix were prepared, differentiated by the addition of PET flakes, amount of activator, and curing temperature. As a reference, the composite with no PET flakes, with 20 wt.% activator, and cured in laboratory conditions at 20 °C was used (20S20). The mix designs that were used are shown in Table 3.

**Figure 2:**
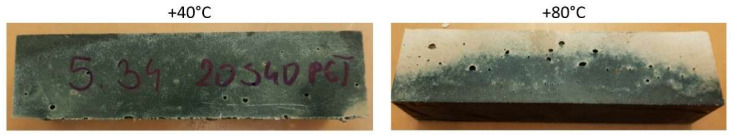
Specimens with 20% activator cured at 40 °C or 80 °C for 12 h.

The mixing procedure consisted of three stages: 1 min of mixing of the binder with the alkali activator and water, followed by 2 min of mixing with the aggregate. Curing at either 40 °C or 80 °C was then applied for 12 h. After casting, the moulds were covered with plastic sheets to prevent loss of moisture. After curing, the temperature was gradually decreased to avoid thermal shock. Samples were removed from the oven when the temperature reached 30 °C. Specimens were left for an additional hour in the moulds in lab conditions and subsequently demoulded. Cured samples showed discoloration, which depended on the applied curing temperature. Specimens cured at a temperature of 40 °C were uniformly green in colour. In the case of specimens cured at a temperature of +80 °C, the green colour disappeared near the top surface of the specimen. Example specimens, photographed at this stage of the curing process, are presented in Figure 2.

For the next 27 days, specimens were sealed in plastic bags and cured at room temperature. After 28 days of curing, the intense green colour had vanished from all specimens. The phenomenon of green coloration of the demoulded specimens is related to the presence of the trisulfur radical anions and disulfur radical anions. Both are green chromophores and can intensify the green coloration of slag-based materials [29].

Specimens prepared for the scanning electron microscope (SEM) examination were cut into small cubes after 28 days of curing. Specimens were immersed in acetone and subsequently put into Branson 1800 Cleaner (Branson Ultrasonics Corporation, Brookfield, CT, USA) to remove impurities. Then, alcohol-evaporated specimens were placed in moulds and impregnated under vacuum with a low-viscosity epoxy resin. Specimens were impregnated under vacuum for one hour. For the next 24 h, specimens were kept at room temperature and then demoulded. To obtain a smooth surface, the specimens were polished using a Struers Labo-Force-100 apparatus (Struers, Ballerup, Denmark). The process of grinding was performed in three steps under a load of 35 kN. Grinding paths were covered with diamond spray with a particle size of 1, 3, and 9 μm. Afterwards, samples were investigated under a JSM-IT100 SEM (JEOL, Ltd., Tokyo, Japan) equipped with a QUAN-TAXEDX (energy-dispersive X-ray spectroscopy) analysis system from BRUKER with ESPRIT 2 software (Bruker Corporation, Billerica, MA, USA). 

All SEM images were taken in the backscattered electron composition (BEC) mode at high vacuum. The accelerating voltage was 10.0 kV and the probe current was 50 mA. The magnification ranged from 50 to 2000 times. The obtained images were subsequently analysed using ImageJ software (LOCI, University of Wisconsin, Madison, WI, USA). PET particles were excluded from the analysis, due to the developing black and white reflections on their surfaces. The threshold value on the grey level histogram was adjusted to extract the targeted features. This enabled us to calculate the total area of all particles, the area of the bulk binder matrix, and the area of microcracks. The particles were divided into aggregates and other particles, which included portlandite and unhydrated GGBFS. 

EDX studies were performed for point analysis. The location of each point was selected in areas corresponding predominantly to the C-S-H based on the grey level of the SEM-BEC images. 

All the tests were conducted using beams with dimensions of 40 mm × 40 mm × 160 mm. Four specimens were prepared for each mix. Firstly, the apparent density was determined. 

The weight of each specimen was measured twice; just after demoulding (after 1 day of curing) and before determining the mechanical properties (after 28 days of curing). The volume of the specimens was measured using a manual calliper with an accuracy of 0.5 mm.

The flexural strength was determined via three-point bending. Sample halves obtained from these tests were used for the compressive strength tests. Three beams were used for the flexural test, and six halves were used for the compressive strength test. The fourth beam was used for the SEM and SEM/EDS analyses. The loading speed was 0.5 mm/min for the flexural strength test and 5.0 mm/min for the compressive strength test. Both strength tests were executed according to UNE EN 1015-11:2001/A1:2007. 

## 3. Results

**Figure 3:**
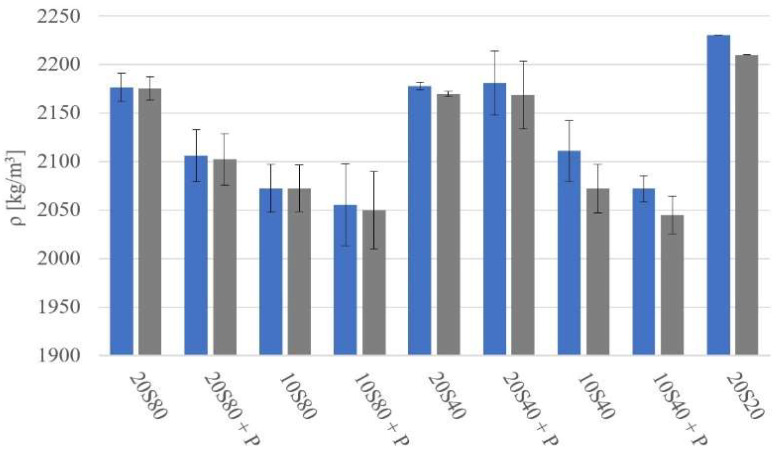
Apparent density of mortars measured after 1 day (blue) and 28 days (grey) of curing.

Before conducting destructive tests, all specimens were weighed and measured. The calculated density (*ρ*) of the mortars after 1 and 28 days of curing is presented in Figure 3. The apparent density of the GGBFS mortars varied from 2050 kg/m^3^ to 2230 kg/cm^3^. Differences in the density values determined after 1 and 28 days of curing were between 1 and 30 kg/m^3^. The curing temperature had no significant effect on the measured density.

**Figure 4:**
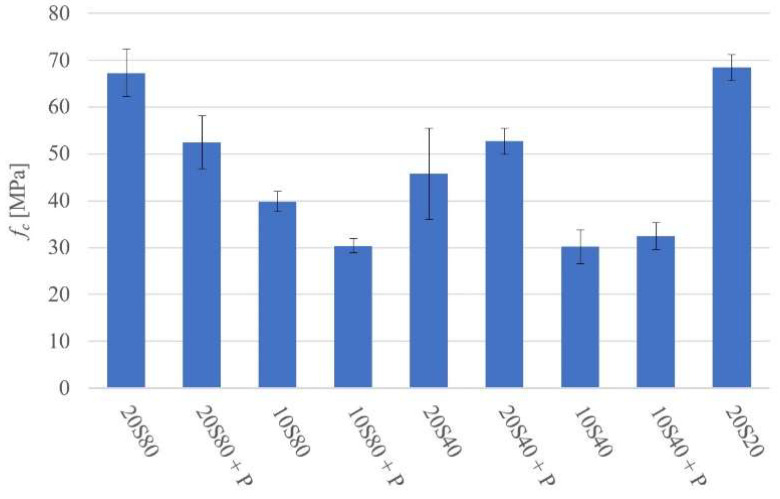
Compressive strength at 28 days.

The compressive strength (*f_c_*) test results are shown in Figure 4. The highest compressive strength values were 68.4 MPa and 67.5 MPa for mixes 20S20 and 20S80, respectively. The lowest strength values were 29.3 MPa, 29.9 MPa, and 31.1 MPa for mixes 10S40, 10S80P, and 10S40P, respectively.

**Figure 5:**
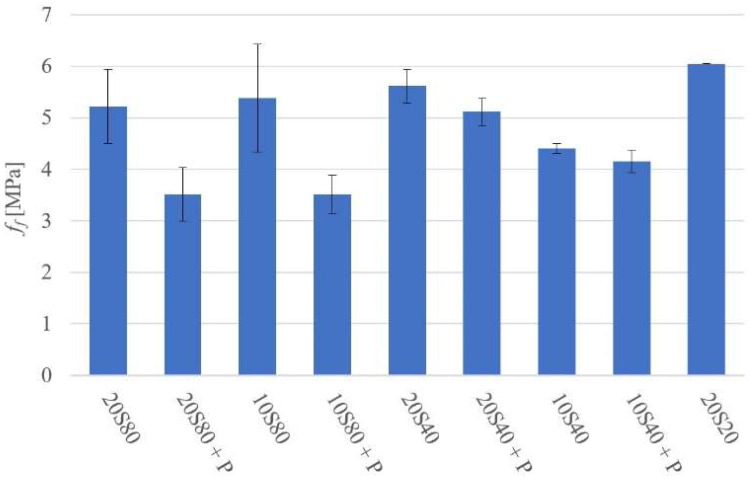
Flexural strength at 28 days.

The 28-day flexural strength (*f_f_*) values varied between 3.5 MPa for mortar 20S80P and 6.1 MPa for mortar 20S20, as shown in Figure 5.

**Figure 6:**
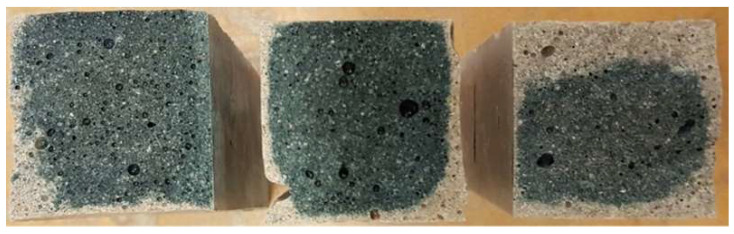
Cross sections of specimens (from left: mortars 20S20, 20S40, and 20S80).

**Figure 7:**
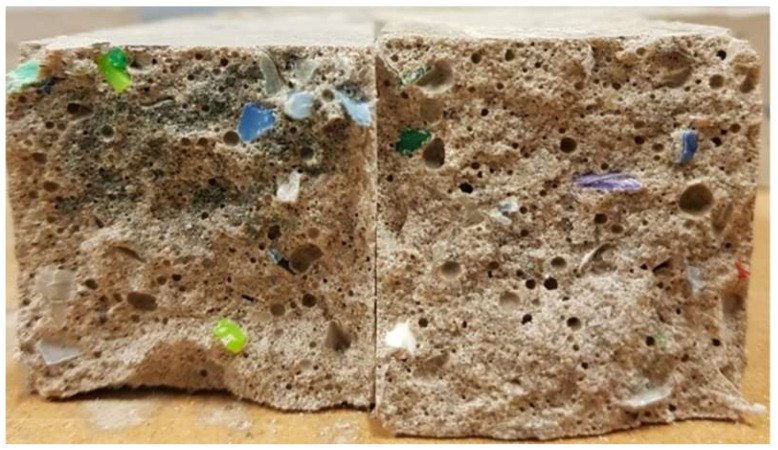
Cross sections of specimens (from left: mortars 10S80P and 10S40P).

Cross sections of the fractured specimens were visually examined to determine the distribution of PET flakes and the microstructure of the binder matrix. The cross sections of fractured samples made of GGBFS mortars that were activated with 20 wt. % of the activator and did not contain PET flakes are shown in Figure 6. The presented samples were heat cured at 20, 40, and 80 °C (20S20, 20S40, and 20S80, respectively). The cross sections of fractured samples made of GGBFS mortars containing 10% activator and PET flakes are shown in Figure 7. Samples were heat cured at either 40 or 80 °C. The observed presence of larger air voids is typical for alkali-activated BFS-based matrixes. The PET flakes were generally uniformly distributed throughout the binder matrix. 

**Figure 8:**
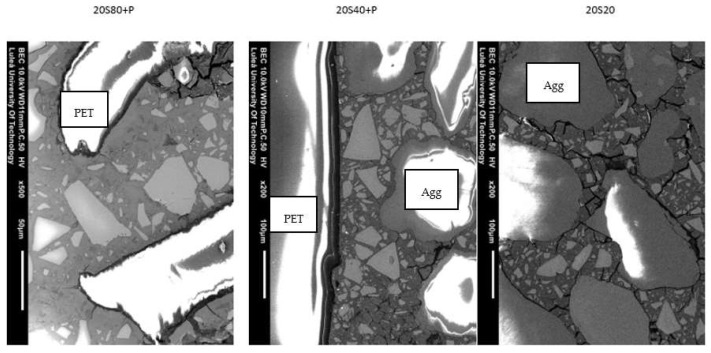
SEM images of mortars 20S20, 20S40 + P, and 20S80 + P.

Examples of SEM-BSE images of GGBFS mortars activated with 20 wt. % solution and exposed to either 20, 40, or 80 °C are presented in Figure 8. 

The results of the EDS analysis are summarised in Table 4. The measurements were taken “in point”. The EDS analysis shows that the structure of GGBFS mortar is non-homogeneous. According to the literature, darker shades of binder matrix indicate a higher concentration of sodium [6]. Moreover, high concentrations of coal and magnesium may suggest that hydrotalcite-like products were formed during the process of hydration (see D0 5198 and D0 5199 in Table 4). Although the analysis of SEM images seems to confirm the results regarding compressive strength, it should also be noted that the obtained results represent only a small extract of a sample.

**Figure 9:**
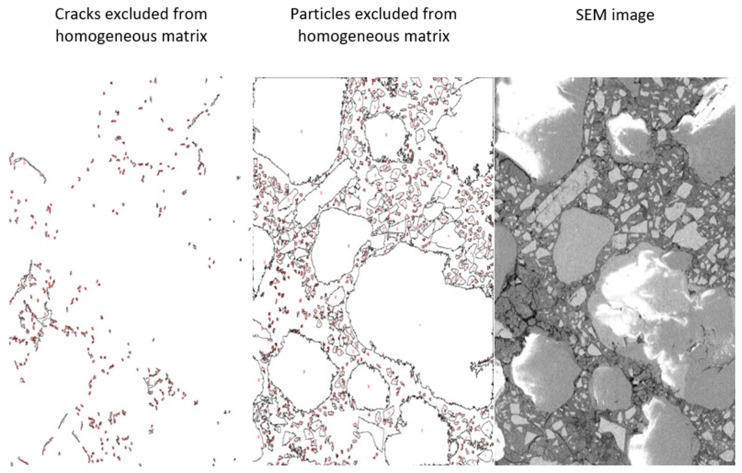
Steps of conducted digital image analysis (20S40).

SEM images were analysed using image processing software (LOCI, University of Wisconsin, USA). The software enabled the exclusion of PET particles and cracks from the area of homogeneous matrix. PET particles were excluded from the analysis, due to the developing black and white reflections on their surfaces. The threshold value on the grey level histogram was adjusted to extract the targeted features. This enabled us to calculate the total area of all particles, the area of the bulk binder matrix, and the area of microcracks. The particles were divided into aggregates and other particles, which included portlandite and unhydrated GGBFS. The stages of the digital processing of images are shown in Figure 9. The computed areas of the homogeneous binder matrix, aggregates, microcracks, and other particles are presented in Table 5.

According to the literature, an increase in curing temperature increases the size and number of pores. The results for samples cured at 40 °C present a lower number of cracks in total than do those for samples cured at 80 °C for similar areas. This is visible in the region of the interfacial transition zone between the aggregate and binder. 

The compressive strength of mortars cured at 40 °C increased when natural aggregate was substituted with PET flakes. The surface attached to PET flakes is denser than the zones near natural aggregate. Therefore, the strength of samples with plastic aggregate decreased when cured at 80 °C. Image analysis showed differences between the sizes of cracks occurring in mortars cured at different temperatures. SEM images showed wider cracks occurring in the transition zone of PET and binder matrix for samples kept at 80 °C for 24 h. The transition zone between artificial aggregate and binder is denser for samples cured at 40 °C, though cracks occurred near natural aggregate. In Table 5, the computed areas from SEM images are presented, showing that a more homogenous binder matrix occurred near PET particles in mortar cured at 40 °C.

## 4. Discussion

The density of the tested mortars tended to be lower for samples incorporating PET, which can be directly related to their lower weight. The most significant decrease was observed for samples activated with 20 wt. % alkali activator and cured at 80 °C. The reason for this result is unknown as PET has shown very good thermal stability at significantly higher temperatures of 150–270 °C. Based on this property, we can exclude the possibility of their de-composition and subsequent creation of additional air voids [29]. Presumably, most measured differences are related to the workability, and thus, to variable amounts of entrapped air. Yet another factor is the commonly observed higher porosity of matrixes cured at elevated temperatures [30].

The highest density, and presumably the lowest porosity, was measured for samples cured at ambient temperature (20S20), which confirmed this trend. The 28-day compressive strength values corresponded to the measured density, with the highest values recorded for the ambient-cured reference sample (20S20). Earlier results also indicated that higher curing temperatures often tend to lower the 28-day compressive strength values of concretes based on sodium-silicate-activated GGBFS [31].

The results indicated that the application of 80 and 40 °C heat curing produced nearly the same ultimate 28-day compressive strength values as when ambient curing was applied. The amount of alkali activator was 20 wt. % in both cases. A lower amount of alkali activator resulted in a lower strength. The presence of PET particles had little effect on the compressive strength in the case of the weaker matrixes. However, the stronger matrixes tended to be weakened more extensively. It can be assumed that the PET acted only as weak inclusions. The flexural strength results confirmed this conclusion (Figure 5). The visual examination of the fractured surfaces showed a relatively uniform distribution of PET in the matrix. The PET particles appeared to be predominantly pulled out of the binder matrix, thus indicating a low bond strength. More detailed SEM and SEM-EDX studies of resin-impregnated and polished samples have generally confirmed these conclusions. Furthermore, the high-temperature heat curing caused severe cracking of the binder matrix (Table 5). This effect is well known and was observed in a number of earlier studies [31,32].

The addition of the PET did not limit the formation of microcracks, which can be interpreted as a lack of a reinforcing effect. The results are also insufficient to assess their possible effect on hydration and depolymerisation processes. However, due to their aggregate-like dimensions, it could be assumed that it is negligible. Furthermore, a possible wall effect could create a weak interfacial transition zone (ITZ) that is porous and rich in portlandite [33]. The SEM study confirmed this assumption by showing less anhydrous GGBFS present close to the PET particles. This indicated a locally higher water-to-cement ratio and a higher degree of hydration. Both factors are typical for the ITZ (Figure 8).

The chemical composition of the binder matrix itself is typical for alkali-activated GGBFS mixes. The atomic Ca/Si ratio varied between 0.5 and 5, which indicated the presence of C-S-H and portlandite. Both phases are typical for Portland-cement-based matrixes and for GGBFS-based geopolymers [34]. The high concentration of magnesium was due to the type of Swedish GGBFS used. Earlier studies also indicated the formation of hydrotalcite-like products [12].

The results of this research are quite promising. Enhancing the strength of mortars provides a basis for further investigation of plastic waste aggregate in alkali-activated mortars. It should be mentioned that in this study, PET flakes were considered as artificial aggregate, whereas in other research, plastics also occur in powdered form [35], as fibres [24,36], pellets [37,38], melted and mixed with sand [39] and GBFS [40] or turned into resin [41]. Moreover, other types of plastics have been investigated as substitutes for natural aggregate, including PVC, LDPE, HDPE, and PP [22,42,43,44,45]. An important issue is that plastics in the form of powder can easily contaminate the environment due to the small size of the grains. Pellet or melted plastic with aggregate requires additional processes during production in comparison to flakes derived from used bottles.

Further research needs to be conducted in order to confirm plastic waste as a valuable substitute for natural aggregate; in particular, the durability needs to be investigated.

## 5. Conclusions

From our results, we drew the following conclusions:

1. Curing at 20 °C and 80 °C produced matrixes with higher density in comparison to mortars cured at higher temperatures; it also resulted in higher compressive strength values.
2. Curing at 40 °C produced matrixes with lower strength values.
3. All tested mortars could be assigned an ordinary concrete strength class ranging from C20/25 to C50/60.
4. The addition of PET generally lowered both the compressive and flexural strength values and introduced an interfacial transition zone with a presumably higher water-to-cement ratio, more portlandite, and less anhydrous GGBFS.
5. Matrixes cured at 80 °C showed more microcracking.
6. The PET particles had no effect on the hydration processes, mainly due to their chemical inactivity and large size, which excluded possible nucleation sites for the hydration to occur.
7. The PET had a very weak bond with the binder matrix, leading to pull-out under tension.

## Figures and Tables

**Table 1:** The use of PET particles in cementitious composites.

References	PET Type	Size [mm]	Amount [%]	Composite
[22]	Crushed	2.36–19	0, 10, 20, 30 vol. of aggregate	Concrete
[23]	Fibres	width < 1, length 8, 16	0, 0.5, 1.0, 1.5, 2.0 mass of mix	Mortar
[24]	Flakes, pellet	1–2	0, 5, 10, 15 vol. of sand	Mortar
[25]	Flakes	1.6–10	3, 10, 20, 50 vol. of sand	Mortar
[26]	Crushed	0.5–5	10, 20, 30, 50 mass of sand	Mortar
[27]	Flakes	0–9.5	5, 10, 15 mass of sand	Concrete

**Table 2:** Chemical composition of GGBFS.

Oxide Type	Content [%]
SiO_2_	37.9
Al_2_O_3_	13.2
Na_2_O	0.5
K_2_O	0.6
CaO	38.5
MgO	7.8
Fe_2_O_3_	0.4

**Table 3:** Mix designs.

Mix	PET Flakes [wt. %]	Activator [wt. %]	Curing Temperature [°C]
20S80	0	20	80
20S80 + P	5	20	80
20S40	0	20	40
20S40 + P	5	20	40
10S80	0	10	80
10S80 + P	5	10	80
10S40	0	10	40
10S40 + P	5	10	40
20S20 (reference)	0	20	20

**Table 4:** Results of EDS analysis.

Spectrum	Carbon	Oxygen	Sodium	Magnesium	Aluminium	Silicon	Calcium	Ca/Si	Al/Si
D0 5198	38.11	52.07		2.10	1.42	4.60	1.69	0.37	0.31
D0 5199	37.49	52.77		1.85	1.28	5.05	1.56	0.31	0.25
D0 5200	28.84	58.89	3.04	0.84	0.67	5.60	2.11	0.38	0.12

**Table 5:** Computed areas from SEM images.

Sample	Binder Matrix [%]	Microcracks [%]	Other Particles [%]	Aggregates [%]
20S40.1	43.0	1.2	32.8	23.0
20S40.2	41.8	3.3	29.7	25.2
20S40.3	42.0	3.1	28.6	26.3
20S80.1	38.7	4.1	30.0	27.2
20S80.2	53.8	4.1	28.7	13.4
20S80.3	75.2	2.3	22.5	0.0
20S40P.1	69.0	1.5	11.4	18.1
20S40P.2	84.7	1.2	14.1	0.0
20S80P.1	54.3	2.4	43.3	0.0
20S80P.2	45.6	6.0	22.0	26.4

## Data Availability

The data presented in this study are available on request from the corresponding author.
